# Assessment of cytotoxicity of (*N*-isopropyl acrylamide) and Poly(*N*-isopropyl acrylamide)-coated surfaces

**DOI:** 10.1186/1559-4106-8-19

**Published:** 2013-08-07

**Authors:** Marta A Cooperstein, Heather E Canavan

**Affiliations:** 1 Department of Chemical and Nuclear Engineering, Center for Biomedical Engineering, University of New Mexico, MSC01 1141, 87131-0001, Albuquerque, NM, USA

**Keywords:** Thermoresponsive polymer, Isopropyl acrylamide, pNIPAM, Cytotoxicity, Plating efficiency, Direct contact test, Concentration gradient, XPS, Goniometry, Mammalian cells

## Abstract

Poly(*N*-isopropyl acrylamide) (pNIPAM) is one of the most popular stimulus-responsive polymers for research. It is especially of great interest in the field of tissue engineering. While it is known that the NIPAM monomer is toxic, there is little conclusive research on the cytotoxicity of the polymer. In this work, the relative biocompatibility of the NIPAM monomer, pNIPAM, and pNIPAM-coated substrates prepared using different polymerization (free radical and plasma polymerization) and deposition (spin coating and plasma polymerization) techniques was evaluated using appropriate cytotoxicity tests (MTS, Live/Dead, plating efficiency). Four different mammalian cell types (endothelial, epithelial, smooth muscle, and fibroblasts) were used for the cytotoxicity testing. The pNIPAM-coated surfaces were evaluated for their thermoresponse and surface chemistry using X-ray photoelectron spectroscopy and goniometry. We found that while cell viability on pNIPAM surfaces decreases when compared to controls, the viability also seems to be deposition type dependent, with sol–gel based pNIPAM surfaces being the least biocompatible. Long term experiments proved that all pNIPAM-coated surfaces were not cytotoxic to the four cell types evaluated in a direct contact test. Plating efficiency experiments did not show cytotoxicity. Cellular sensitivity to pNIPAM and to the NIPAM monomer varied depending on cell type. Endothelial cells consistently showed decreased viability after 48 hours of exposure to pNIPAM extracts and were more sensitive than the other cell lines to impurities in the polymer.

## Background

Poly(*N*-isopropyl acrylamide) (pNIPAM) is a thermoresponsive polymer that undergoes a phase change in a physiologically relevant temperature range. Mammalian cells can be easily cultured on pNIPAM at 37°C. When the temperature is lowered to below pNIPAM’s lower critical solution temperature (LCST, 32°C), the polymer’s chains extend and cells detach in intact sheets [1, 2]. This detachment method is preferred to enzymatic digestion or mechanical scraping [3–5]. Mechanical scraping can result in broken cell sheets and destroyed cells, while enzymatic digestion does not preserve the cell sheet, breaking it into single cells. Temperature-dependent lift-off results in an intact cell sheet that can readily attach to another surface. This non-destructive release of cells opens up a wide range of applications, including the use of pNIPAM for tissue engineering, for controlling bioadhesion and bioadsorption, and for manipulation of microorganisms. These uses are summarized in our recent article [6].

PNIPAM is one of the most commonly used stimulus-responsive polymers for research [7, 8]. There is currently a great deal of research regarding the development of engineered tissues or devices using pNIPAM [9–12]. Many of these devices will ultimately be used on humans. However, there has been relatively little conclusive research regarding the extent of its cytotoxicity or biocompatibility [13–19]. The International Organization for Standardization (ISO) requires extensive testing of medical devices, with *in vitro* cytotoxicity being one of the required assessments [20].

It has previously been demonstrated that the NIPAM monomer is toxic [21]. There are conflicting opinions, however, as to whether the polymerized form of NIPAM (pNIPAM) is toxic. One reason for this conflict is because there are very few publications (less than 15 studies) [13–19, 22–27] that explore the cytotoxicity of pNIPAM, as compared to hundreds of publications using pNIPAM for cell-based research. None of the studies are comprehensive. Instead, they focus on isolated cell lines (e.g., only fibroblasts [18], smooth muscle cells [28], or endothelial cells [19]), and employ different methods of cytotoxicity testing (e.g., morphologic observations [15], concentration gradients [13], or direct contact test [14]). While some of the studies examine pNIPAM without any additives, others concern copolymers of pNIPAM [28], or other forms such as hydrogels [14] or nanoparticles [16] that are composed not only of pNIPAM but also of other compounds. Such compounds could contribute to the cytotoxicity, or even be the single source of cytotoxicity of the composite product. Only a few of these studies investigate the cytotoxicity of pure pNIPAM [13–19]. Of these studies, not one investigated more than a single polymerization technique, although various polymerization and deposition techniques are used to generate pNIPAM surfaces for cell sheet engineering. In addition, these studies examined different forms of pNIPAM, such as hydrogels [14], nanoparticles [15, 16], or pNIPAM in solution [13, 17–19].

It is critical to investigate the cytotoxicity of pNIPAM not only at body temperature, but also below pNIPAM’s LCST, as temperature may affect the cytotoxicity of the polymer. Only one of the above mentioned studies [13] investigated the cytotoxicity of pNIPAM above and below its LCST; Vihola, et al., showed that there is lower cellular viability above the LCST of the polymer. The remaining studies yielded contradictory results, including no significant cytotoxicity found [16], different cell viability depending on cell type [15], lower cell viability in the presence of lower concentrations of pNIPAM [13, 18, 19], and lower cell viability in the presence of higher pNIPAM concentrations [17]. None of these studies investigated the effects of growing cells directly on pNIPAM-coated surfaces or the effect of pNIPAM fragments that may leach out of the surface into the cell culture medium.

In this work, we examine the cytotoxicity of the pNIPAM monomer, pNIPAM, and pNIPAM films. PNIPAM was synthesized using free radical polymerization (frpNIPAM), as this is one of the most commonly used methods for the synthesis of pNIPAM for cytotoxicity studies [13–16, 18]. Commercially available pNIPAM was also used for the experiments (cpNIPAM). PNIPAM films were generated using vapor-phase plasma polymerization of NIPAM (ppNIPAM), spin-coating of cpNIPAM/tetraethyl orthosilicate sol gel (spNIPAM), spin-coating of cpNIPAM dissolved in isopropanol (cpNIPAM/IPA), and spin-coating of frpNIPAM, also dissolved in isopropanol (frpNIPAM/IPA). These techniques alone account for the majority of the ongoing research in this area (~90%). The cytotoxicity of NIPAM and pNIPAM was assessed using four different cell lines: endothelial cells, epithelial cells, smooth muscle cells, and fibroblasts. pNIPAM’s toxicity was assessed in two ways: by direct contact with the cells and by testing pNIPAM extracts.

## Materials

PNIPAM (molecular weight of ~40,000) was purchased from Polysciences, Inc. (Warrington, PA). *N*-isopropyl acrylamide (99%), and diethyl ether (99.5%, extra dry) were purchased from Acros Organics (Geel, Belgium). 2,2’-azobis(2-methylpropionitrile) (AIBN, 98%), tetratethyl orthosilicate (TEOS), dioxane (99.8%, anhydrous), and isopropanol were purchased from Sigma Aldrich (St. Louis, MO). The 200 proof ethanol, HPLC-grade methanol, HPLC-grade dichloromethane, HPLC-grade acetone, and hydrochloric acid (1 normal) were purchased from Honeywell Burdick & Jackson (Deer Park, TX).

Round glass cover slips (15 mm) were purchased from Ted Pella, Inc. (Redding, CA). Square glass cover slips were purchased from Fisher Scientific (Pittsburgh, PA). The silicon chips were obtained from Silitec (Salem, OR).

Dulbecco’s modified eagle’s medium (DMEM), minimum essential medium with alpha modification (αMEM), and Dulbecco’s phosphate buffered saline without calcium or magnesium were purchased from HyCLone (Logan, UT). Bovine aortic endothelial cells (BAECs) were from Genlantis (San Diego, CA). Smooth muscle cells (CRL-1444, SMCs), fibroblasts (MC3T3-E1, 3T3s) and Vero cells (CCL-81) were obtained from ATCC (Manassas, VA). Fetal bovine serum (FBS) and penicillin/streptomycin were from HyCLone (Logan, UT). Minimum Essential Medium Non-Essential Amino Acids solution (MEM NEAA) and 0.25% trypsin/EDTA were purchased from Gibco (Grand Island, NY). CellTiter 96® AQueous One Solution Cell Proliferation Assay (MTS) was obtained from Promega (Madison, WI). LIVE/DEAD viability kit was purchased from Invitrogen (Grand Island, NY).

## Methods

### Surface preparation

For surface analysis, silicon wafers were cut into 1 cm × 1 cm squares for X-ray photoelectron spectroscopy (XPS), and 0.8 cm × 3 cm rectangles for goniometry. The surfaces were cleaned in an ultrasonic cleaner from VWR International (West Chester, PA) twice in each of the following solutions for 5 minutes: dichloromethane, acetone, and methanol.

Glass cover slips were cleaned for 30 min with an acid wash (a 1:1 solution by volume of methanol and hydrochloric acid), rinsed with deionized water, and dried with nitrogen.

### Plasma polymerization (ppNIPAM)

Plasma polymerization was performed in a reactor chamber fabricated to our design specifications by Scientific Glass (Albuquerque, NM) following a method previously described [29]. To spark a plasma in the chamber, two 2.5 cm copper electrodes were connected to a Dressler (Stolberg, Germany) matching network and Cesar radio frequency (rf) power generator from Advanced Energy (Fort Collins, CO). Argon etching (40 W, 2 min) and methane adhesion-promoting layer (80 W, 5 min) were performed before pNIPAM deposition. During pNIPAM deposition, the power setting of the rf generator was slowly decreased from 100 W to 0 W (100 W for 5 minutes, 10 W for 5 minutes, 5 W for 5 minutes, 1 W for 10 minutes, followed by 10 minutes at 0 W). After the samples were removed from the reactor chamber, they were rinsed with cold deionized water to remove any uncross-linked monomer, dried with nitrogen, placed in a Petri dish and sealed with Parafilm under nitrogen.

### Free radical polymerization of NIPAM (frpNIPAM)

Free radical polymerization of NIPAM was adapted from Vihola et al [13]. Briefly, the monomer (133 mmol) was dissolved in 55 mL of dioxane. The polymerization solution was degassed with nitrogen and heated to 70°C. Once the desired temperature was reached, the solution of initiator [AIBN (0.1%, 0.133 mmol) in 5 mL of dioxane] was added to the polymerization solution. The reaction was allowed to proceed for 18 hours. After 18 hours, the polymerization solution was cooled to room temperature and the polymer was precipitated into excess cold diethyl either twice. The resulting powder was dried in a vacuum oven overnight.

### SpNIPAM solution preparation

Solution preparation using sol–gel (spNIPAM) was performed following a method previously described [30]. Briefly, 35 mg of pNIPAM, 5 mL of deionized water, and 200 μL of hydrochloric acid were mixed and a weight percentage of pNIPAM was determined. In a separate container, 250 μL of TEOS solution (1 TEOS : 3.8 ethanol : 1.1 water : 0.0005 HCl), 43 μL of deionized water, and 600 μL of ethanol were mixed and weighted. The appropriate amount of the pNIPAM solution was added to achieve the final weight percentage of pNIPAM of 0.35%.

### SpNIPAM solution deposition

100–250 μL of the spNIPAM solution was evenly distributed onto clean glass cover slips and Si chips placed on a spin coater, model 100 spinner from Brewer Science, Inc. (Rolla, MO). The surfaces were spun at 2000 rpm for 60 seconds. The surfaces were stored under nitrogen in a Parafilm covered Petri dish until used for cell culture or surface analysis.

### Deposition of FrpNIPAM/isopropanol and CpNIPAM/isopropanol

FrpNIPAM or cpNIPAM were dissolved in isopropanol to achieve 1% of pNIPAM by weight. The solutions were the spun onto surfaces in the same manner as the spNIPAM surfaces.

### Nuclear magnetic resonance (NMR)

The NMR spectra of frpNIPAM and cpNIPAM were taken with an Avance III NMR spectrometer (Bruker, Billerica, MA). It is a 300 MHz, standard bore, nanobay instrument. Spectra were obtained on a 5 mm broadband/proton probe, at room temperature, using CDCl3 as a solvent.

### Size exclusion chromatography

Size exclusion chromatography (SEC) analyses were performed in chloroform with 0.5% (v/v) triethylamine (1 mL/min) using a Waters Breeze system equipped with a 2707 autosampler, a 1515 isocratic HPLC pump and a 2414 refractive index detector. Two styragel columns (Polymer Laboratories; 5 μm Mix-C), which were kept in a column heater at 35°C, were used for separation. The columns were calibrated with polystyrene standards (Varian).

### X-ray photoelectron spectroscopy

Survey spectra of the pNIPAM surfaces were taken at the National ESCA and Surface Analysis Center (NESAC/BIO) using Kratos Axis-Ultra DLD (Manchester, UK) and Surface Science Instruments S-probe spectrometers. Both instruments use monochromatized Al Kα X-rays, low-energy electron flood gun for charge neutralization, and were operated in low (10^-9^ Torr) pressure. The analysis area was < 800 μm. Data analysis was carried out using the appropriate analysis programs. The binding energy scales of the high resolution spectra were calibrated by assigning the most intense C1s high resolution peak a binding energy of 285.0 eV. A linear function was used to model the background.

### Goniometry

Contact angle measurements were performed with an Advanced Goniometer model 300-UPG from ramé-Hart Instrument Co. (Mountain Lakes, NJ) with an environmental chamber and the DROPimage Standard program. Inverted bubble contact angles were taken in Millipore water (18 MΩ). Angles were obtained at room temperature (21°C) and at body temperature (37°C) using the Temp Controller model 100–500 connected to the environmental chamber.

### Cell culture

For cell culture, BAECs, SMCs, and Vero cells were cultured according to previously established protocols [30] in DMEM supplemented with 10% FBS, 1% penicillin/streptomycin. For BAECs, 1% MEM NEAA was also added. 3T3s were cultured in αMEM supplemented with 10% FBS and 1% penicillin/streptomycin. Cells were incubated at 37°C in a humid atmosphere with 5% CO_2_. When confluent, the cells were washed with 0.25% trypsin/EDTA (DPBS for BAECs) and lifted from cell culture flasks with 0.25% trypsin/EDTA.

### Cytotoxicity testing

All cytotoxicity experiments (except for plating efficiency) were performed in 5% FBS media according to ISO standards [20]. Media without phenol red was used for experiments evaluated with the MTS assay, as the dye contributes to increased background absorbance [31].

### Direct contact test

For the direct contact test, cells were seeded directly on spNIPAM, frpNIPAM, cpNIPAM, and ppNIPAM surfaces (24-well TCPS plate, 20,000 cells/well) in a regular cell culture media. Morphological observations, MTS assay, and LIVE/DEAD assay were performed after 48 and 96 hours of cell culture. The procedure for LIVE/DEAD assay was adapted from the procedure supplied by the manufacturer [32]. To create combined LIVE/DEAD solution, 1 μL of the Calcein solution (to stain live cells) and 1 μL of the ethidium solution (to stain dead cells) were added per 1 mL of DPBS. Fluorescent images were taken on a Nikon Eclipse TS200F inverted microscope with an epi-fluorescence attachment (Nikon Instruments, Melville, NY) and a SPOT Insight color mosaic digital camera (Diagnostic Instruments, Sterling Heights, MI).

### Preparation of extracts

Extracts from ppNIPAM, spNIPAM, cpNIPAM, and frpNIPAM were obtained at room (20°C) and body (37°C) temperature. To make extracts, a pNIPAM surface was incubated in regular cell culture media (surface to liquid volume ration of 1.5 cm^2^/mL) for 24 hours at room and body temperature. After 24 hours, the resulting extracts were transferred to a centrifuge tube and kept in a refrigerator for experiments with cells.

### Plating efficiency

The above mentioned extracts were used for plating efficiency assay. The assay was performed according to the method developed by Ham and Puck [33]. Briefly, 200 cells were seeded in a round Petri dish containing 5 mL of the extracts or 5 mL of regular cell culture media (control). Cells were left in an incubator for an amount of time that allowed them to double ten times (that time was determined based on the doubling time of the specific cell line). After the required amount of time cells were fixed and stained using Carnoy’s fixative (3:1 methanol : acetic acid by volume, 0.5% crystal violet by weight). The colonies formed on the dish were counted and compared to the colonies formed on the control. The plating efficiency was calculated using the following equation:
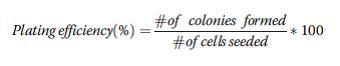


### Extracts study

To perform experiments with extracts, 8000 cells were seeded per well in a 96-well plate. After 24 hours in regular cell culture media, the media were replaced with extracts in 3 concentrations (1% extracts in regular media, 10%, and 100% extracts). MTS assay was performed after 24 and 48 hours. For the MTS assay, 20 μL of MTS solution were added to each well. After 3 hours of incubation at 37°C, absorbance readings were measured on a SpectraMax M2 plate reader (Molecular Devices, Sunnyvale, CA) at 490 nm.

### Concentration gradient

For concentration gradient experiments, frpNIPAM and cpNIPAM were dissolved in tissue culture media in the concentrations of 0.1, 0.5, 1, 2, 3, 4, 5, 6, 7, 8, 9, and 10 mg/mL. Cells were seeded in 96-well tissue culture polystyrene (TCPS) plates at the concentration of 8000 cells/well. Cells were cultured in the presence of the regular cell culture media for 24 hours. After 24 hours, the culture media was replaced with the test solution (pNIPAM dissolved in media). Morphology observations and MTS assay were performed after 24 and 48 hours of exposure to the test solutions.

### Statistical analysis

Statistically relevant data were obtained by replicating all procedures three times. Each replication of the experiment utilized three surfaces, with each surface analyzed in three different sites across the surface. This method was used for both surface analysis and cell behavior studies. The results are expressed as average values ± SEM. Excel’s ANOVA function and a student *t*-test were used to verify statistical relevance, with significance established at p<0.05.

## Results and discussion

### Polymerization and surface preparation

Free radical polymerization is one of the most commonly used methods for the synthesis of pNIPAM for cytotoxicity studies [13–16, 18]. Therefore, in addition to performing cytotoxicity experiments with NIPAM monomer and commercially available pNIPAM (cpNIPAM), pNIPAM was synthesized by free radical polymerization using AIBN. The resulting polymer (frpNIPAM) was examined using nuclear magnetic resonance (NMR) spectroscopy to confirm successful polymerization.

**Figure 1:**
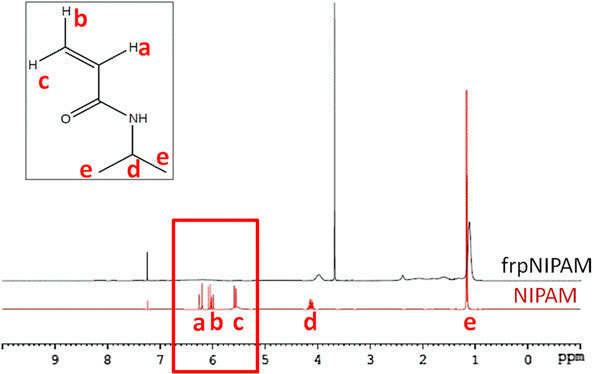
**NMR spectrum for frpNIPAM and NIPAM.** Inset shows chemical structure of the NIPAM monomer. Hydrogens bound to alkenes are indicated as “**a**”, “**b**”, and “**c**” in the inset and spectrum.

Figure 1 shows two NMR spectra: the frpNIPAM polymer (top, in black), and the NIPAM monomer (bottom, red). Highlighted in the box is the region between 5.5 and 6.5 ppm, in which peaks for hydrogens adjacent to double bonded carbons usually appear. These peaks are clearly visible in the spectrum of the monomer, as NIPAM has 3 hydrogens adjacent to two carbons joined with a double bond (see them labeled with a, b, and c on the inset in Figure 1 of the chemical structure of NIPAM and on the NMR spectra). These peaks are, however, missing from the NMR spectrum of the frpNIPAM. The disappearance of these peaks indicates successful formulation of the polymer.

To confirm polymerization of frpNIPAM, the polymer was further tested using size exclusion chromatography. The weight-average molecular weight of frpNIPAM was found to be 104,000 (data not shown), with a polydispersity index of 1.89. FrpNIPAM has a higher molecular weight than cpNIPAM, the other pNIPAM polymer used for testing in this study, which is reported to have a molecular weight of approximately 40,000.

**Figure 2:**
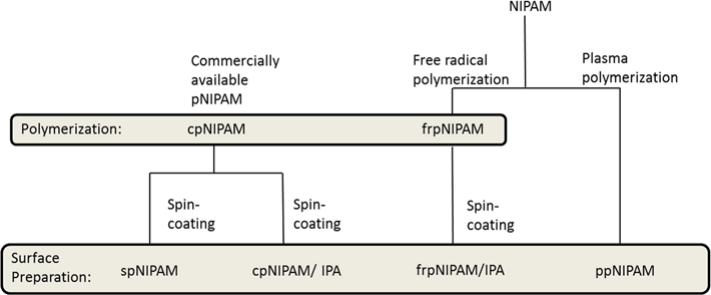
Overview of polymerization and surface preparation techniques used in this manuscript.

PNIPAM films were generated using vapor-phase plasma polymerization of NIPAM (ppNIPAM) [29], spin -coating of cpNIPAM/tetraethyl orthosilicate sol gel (spNIPAM) [30], spin-coating of cpNIPAM dissolved in isopropanol (1 wt% cpNIPAM/IPA), and spin-coating of frpNIPAM, also dissolved in isopropanol (1 wt% frpNIPAM/IPA). These techniques account for the majority of the ongoing research in this area (estimated ~90% of number of publications). Figure 2 shows schematically how the surfaces were generated. Commercially available pNIPAM was used to make spNIPAM and cpNIPAM/IPA surfaces. The NIPAM monomer was used to directly generate plasma polymerized surfaces (ppNIPAM) as well as frpNIPAM, which was in turn used for generation of frpNIPAM surfaces. Overall, four different types of pNIPAM-coated surfaces were used for the testing of pNIPAM’s cytotoxicity (ppNIPAM, spNIPAM, cpNIPAM/IPA, and frpNIPAM/IPA), and two pNIPAM formulations were used for concentration gradient experiments (frpNIPAM and cpNIPAM). For more detail about ppNIPAM and spNIPAM surfaces please see our earlier publications [29, 30, 34].

### Surface chemistry

**Figure 3:**
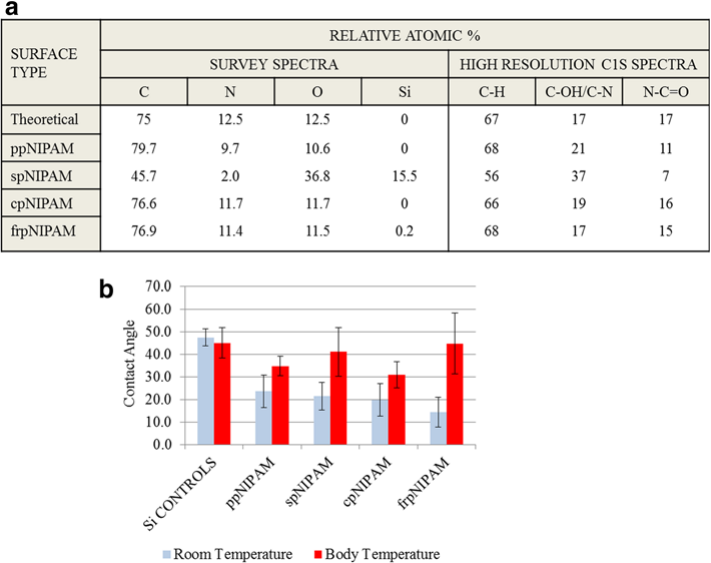
**XPS and contact angle results.a)** Elemental composition of pNIPAM-coated surfaces from XPS data analysis; **b)** Contact angles of pNIPAM-coated surfaces measured at room and body temperature. N=9 with a standard deviation of ±1, except for spNIPAM with standard deviation of ±7.

The surface chemistry of these pNIPAM-coated surfaces was assessed by X-ray photoelectron spectroscopy (XPS). Figure 3a shows the results of survey and high resolution C1s spectra for all four types of surfaces. The first row of data shows the expected values (“Theoretical”) as calculated from the stoichiometry of the monomer. An additional column for silicon (Si) was added to the table as spNIPAM contains Si due to the TEOS solution. In addition, since the pNIPAM was coated on Si wafers, the presence of Si could indicate that pNIPAM films showing Si peaks are ≤50 nm thick. PpNIPAM, cpNIPAM, and frpNIPAM surfaces have elemental composition consistent with that predicted from the monomer structure (~75% C, 12.5% O, and 12.5% N). However, spNIPAM surfaces’ composition differs significantly from the theoretical composition (45.7% C, 36.8% O, 2% N, and 15.5% Si). The high standard deviation of the XPS data indicates that the spNIPAM surfaces did not have an even surface coverage. The XPS analysis revealed a large percentage of either TEOS or underlying surface exposed (Si accounting for 15.5% of elemental composition), which most likely resulted from pNIPAM precipitating out of the sol gel during the deposition. FrpNIPAM surfaces also show a small percentage of surface exposed (0.2% of Si present in the survey spectrum). Examination of the data showed that this variation occurs from spot to spot, not from sample to sample, and most of the surface was still covered with pNIPAM coating. For high resolution C1s spectra for all four types of surfaces see Additional file 1: Figure S1.

### Polymer thermoresponse

The thermoresponse of the pNIPAM-coated surfaces was examined by goniometry. Inverted bubble contact angles were taken at room temperature (20°C) and at body temperature (37°C). Figure 3b shows the results of these measurements. The controls (Si chips) did not show any thermoresponse, with both average values at room temperature (blue) and body temperature (red) at ~45°C. In comparison, pNIPAM-coated surfaces showed thermoresponse. Although the values differ for different preparation techniques, all surfaces displayed thermoresponse with contact angles at body temperature larger than those at room temperature, which is the desired result for surfaces coated with pNIPAM [35]. The large standard deviations for spNIPAM and frpNIPAM are much larger (26 and 30% for spNIPAM and frpNIPAM respectively at body temperature) than those of ppNIPAM and cpNIPAM (13 and 19%), indicating that spNIPAM and frpNIPAM yield substrates with more spot-to-spot variability, confirming our XPS observations.

### Cytotoxicity experiments

All four types of pNIPAM-coated surfaces were used for cytotoxicity studies with four different cell types: bovine aortic endothelial cells (BAECs), monkey kidney epithelial cells (Veros), rat aorta smooth muscle cells (SMCs), and fibroblasts (3T3s). In addition to pNIPAM-coated surfaces, we also tested the NIPAM and pNIPAM powders, without tethering them to a surface.

### Cytotoxicity of the NIPAM monomer

Cytotoxicity of the monomer was tested using an MTS assay, which tests mitochondrial activity in live cells [31]. Table 1 shows the results of cytotoxicity experiments with the monomer. It was previously reported that the monomer shows cytotoxicity effects at concentrations from 0.5 mg/mL, with cellular viability decreasing with increasing concentration of the monomer [13]. For this study, NIPAM was dissolved in cell culture media at the concentration of 5 mg/mL, and tested with the above mentioned four cell types. The compound is considered cytotoxic if cellular viability after exposure to the compound is below 70% [20].

**Table 1:** MTS assay results of the cytotoxicity experiments for all four cell types after 24 and 48 hours of exposure to the NIPAM monomer

	% Viability			
	24 hours of exposure		48 hours of exposure	
	Average	Standard deviation	Average	Standard deviation
BAECS	38	3	18	2
Veros	32	3	16	9
SMCs	59	16	36	13
3T3s	**82**	6	48	4

Bold indicates viability above 70%.

All cell types showed reduced viability after 24 and 48 hours of cell culture in the presence of the monomer solution. 3T3s showed the most resistance to the toxic effects of NIPAM, with cell viability of slightly above 80% after 24 hours of exposure (at the concentration of 5 mg/mL, bold in Table 2). After 48 hours however, the viability of 3T3s decreased to below 70% (to 48%). The remaining cell types had significantly lowered viability after 24 hours, and this viability decreased even more after 48 hours of exposure. Therefore, although the monomer proved to be cytotoxic to all tested cell types, the extent to which it is toxic to cells at the concentration tested in this study depended on the cell type: the endothelial (BAECs) and epithelial (Vero) cells were the most sensitive to the monomer, whereas the fibroblasts were the most resistant.

**Table 2:** Plating efficiency results for BAEC, Vero, SMC, and 3T3 cells exposed to the NIPAM monomer and extracts from ppNIPAM, spNIPAM, cpNIPAM, and frpNIPAM

Type of extracts	Plating efficiency			
	BAECs	Veros	SMCs	3T3s
NIPAM	0	0	0	0
ppNIPAM (20°C)	98	94	95	96
PPNIPAM (37°C)	103	93	89	91
spNIPAM (20°C)	100	96	100	101
spNIPAM (37°C)	105	91	93	**82**
cpNIPAM (20°C)	102	94	88	97
CPNIPAM (37°C)	102	90	91	**78**
frpNIPAM (20°C0)	97	91	93	91
frpNIPAM (37°C)	108	90	98	**88**

Bold indicates extracts with decreased viability at 37°C.

### Cytotoxicity of pNIPAM-coated substrates

The cytotoxicity of pNIPAM-coated surfaces was evaluated in three different ways: by direct contact test, plating efficiency, and by an MTS assay evaluating cellular viability after cell culture in the presence of pNIPAM extracts [20, 33]. Direct contact tests indicate how cells respond to being cultured directly on pNIPAM-coated surfaces, as opposed to plating efficiency and extracts, which test cellular response to pNIPAM in a more indirect manner.

**Figure 4:**
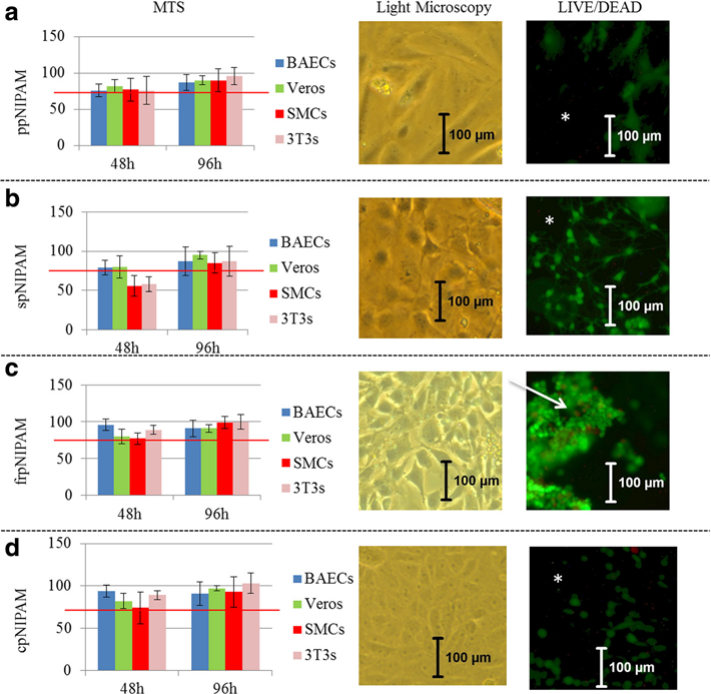
**Direct contact test results.** MTS assay results (first column) for all four cell types after cell culture for 48 and 96 hours on **(a)** ppNIPAM surfaces, **(b)** spNIPAM surfaces, **(c)** frpNIPAM surfaces, and **(d)** cpNIPAM surfaces. Light microscopy (second column) and LIVE/DEAD assay results (third column) for **(a)** SMCs after 96 hours of culture on ppNIPAM surfaces, **(b)** 3T3s after 96 hours of culture on spNIPAM surfaces, **(c)** BAECs after 96 hours of culture on frpNIPAM surfaces, and **(d)** Veros after 96 hours of culture on cpNIPAM surfaces. Red line indicates the viability of 70%, below which a compound is considered to be cytotoxic. Asterisks point to the exposed surfaces from which cells have detached (in black). The arrow points to a sheet of detached, live cells.

Direct contact testing consists of cells being cultured directly on the pNIPAM-coated surfaces [20]. Briefly, cells were cultured on the surfaces for up to 96 hours. Figure 4 shows cell viabilities after 48 and 96 hours of cell culture on ppNIPAM (a), spNIPAM (b), frpNIPAM (c), and cpNIPAM (d). The first column shows the MTS assay results for all four cell types, after these cells were cultured on pNIPAM coated surfaces. For ppNIPAM (a), frpNIPAM (c), and cpNIPAM (d) surfaces, all cell types showed viability of ≥70% for both time points. SMCs and 3T3s showed significantly lower viability (below 70%) after 48 hours of culture on spNIPAM surfaces when compared to BAECs and Veros. However, after 96 hours, cellular viability is comparable to the other surfaces (at ~90%). It appears, that initial attachment and proliferation of 3T3s and SMCs is hindered on spNIPAM surfaces, indicating these cells may be more sensitive to the surface chemistry and topography differences found using XPS and goniometry.

Morphological observations (second column, cells shown after 96 hours) revealed cells with normal morphology, spreading and growing to confluency on all four types of surfaces. However, when seeded on spNIPAM surfaces, cells first appeared to attach to the exposed glass surface, not to the pNIPAM coating. The uneven coverage on the surfaces, precipitation of pNIPAM from the sol gel solution observed at some spots on the surfaces, and the possibility of the presence of traces of other materials on the surface (such as ethanol used for sol gel process) are likely to result in surfaces that do not promote cell adhesion. Overall, there were fewer cells attached to spNIPAM surfaces after 24 hours than to the other three types of surfaces. This could explain lower values of viability after 48 hours. After 96 hours, cells that did attach to the surface had enough time to divide, resulting in higher viability values.

The third column of Figure 4 shows the results of a LIVE/DEAD assay on the four types of surfaces. Cells attached to the surfaces stained green, meaning that they were alive. As the LIVE/DEAD assay requires incubation at room temperature, most of the cells detached from the surfaces leaving exposed black pNIPAM surfaces (indicated by the asterisks in Figure 4). This detachment was expected and desired, as it proves that these surfaces are thermoresponsive. A detached, wrinkled sheet of BAECs can be seen in Figure 4 (c) (indicated with a red arrow). There were a few red stained (dead) cells visible on some of the images taken during the test. However, controls (uncoated glass slides) also showed a small percentage of dead cells after staining (see Additional file 2: Figure S2). There was no difference in the ratio of dead cells to live cells between the controls and test surfaces. Therefore, it can be concluded that there were no cytotoxic effects found for the surfaces and cell types evaluated in this experiment.

### Extracts

The pNIPAM-coated surfaces were used to generate pNIPAM extracts, which were then used for cytotoxicity testing. One of the cytotoxicity tests performed was plating efficiency. This is a very sensitive test, as isolated cells do not have their neighbors to shield them from potentially harmful compounds present in the cell culture media [33]. The controls, cells cultured in regular cell culture without pNIPAM extracts, are under optimal conditions. If there is anything in the pNIPAM extracts that prevents the cells from proliferating, the percent plating efficiency would be decreased when compared to controls.

Table 2 shows the results of this test for all four cell types and for all four types of surfaces. The extracts were made at two different temperatures, 37 and 20°C, to test if the temperature has any influence on what (if anything) leaches off the surface into the surrounding media. It is important to note that larger amounts of polymer are expected to be found in the extracts generated at room temperature, since the polymer films are not covalently bound to the surfaces. As expected, no colonies were formed in the presence of 5 mg/mL of NIPAM in the media, verifying that the NIPAM monomer is cytotoxic. The remaining extracts did not result in significant decrease of plating efficiency for BAECs, Veros, or SMCs.

3T3s showed a slightly decreased plating efficiency for cells exposed to spNIPAM, frpNIPAM, and cpNIPAM extracts generated at 37°C (~10%) when compared to the same extracts generated at room temperature. This effect did not occur for ppNIPAM surfaces. This is most likely because ppNIPAM surfaces are the only physically grafted surfaces tested in this study, and consequently, are likely to be the most stable surfaces. Statistical analysis revealed that there is a significant difference in plating efficiencies for cpNIPAM surfaces between 20 and 37°C, and for spNIPAM surfaces between these two temperatures, with lower plating efficiencies values for extracts obtained at 37°C. SpNIPAM surfaces showed lower initial attachment for 3T3s during the direct contact test, therefore, it is possible that it is the inhospitable surface chemistry of these surfaces that obstructs initial cell attachment and growth. It is important to mention, that these values are still above the 70% cytotoxicity cut off; thus, although the results are significant, the lowered values do not render these surfaces cytotoxic.

The extracts from the pNIPAM coated surfaces were further evaluated by first growing cells on uncoated TCPS for 24 hours with regular media, and then changing the media for extracts. Three extracts concentrations were used: 100%, 10% (10% extracts, 90% regular media), and 1%. Experiments on epithelial cells, smooth muscle cells, and fibroblasts did not show any drop in cellular viability for any of the extracts concentrations or time points. See Additional file 3: Figure S3, Additional file 4: Figure S4, Additional file 5: Figure S5 for these results. However, BAECs consistently showed decreased cell viability for all eight types of extracts after 48 hours of exposure at the 100% concentration.

**Figure 5:**
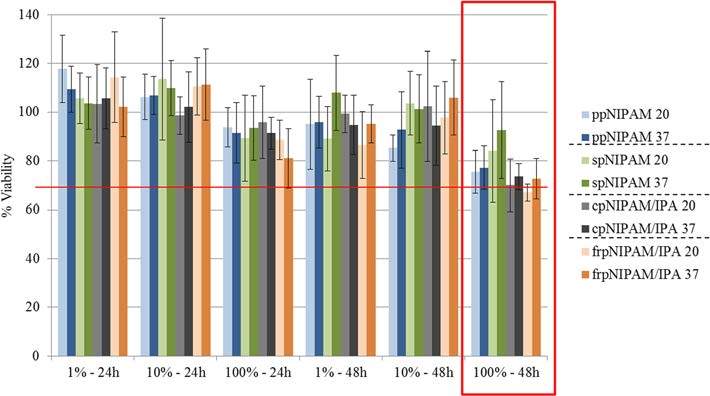
**MTS assay results for culture of BAECs in the presence of pNIPAM extracts.** Red line indicates viability of 70%, below which a compound is considered to be cytotoxic. Red box indicates the only time and concentration for which the viability of BAECs was lowered to ≤ 70% across all pNIPAM coated surfaces. Corresponding figures for Veros, SMCs, and 3T3s can be found in supplemental information.

Figure 5 shows the results for all concentrations, time points, and types of extracts for BAECs. It is immediately visible, after 24 hour exposure the 1 and 10% concentrations do not affect the viability, as the viabilities are all ~100%. The average viabilities drop slightly after 24 hours of exposure to 100% extracts. However, as the assay results are still at or above 80%, they are still considered not cytotoxic. Forty eight hours of exposure at 1 and 10% did not result in a significant drop of viabilities (although the average viabilities are lower than the corresponding viabilities after 24 hours). The only time and concentration for which the viabilities of BAECs were lowered to about (or below 70%) was 100% extracts at 48 hours of exposure (red box in Figure 5). None of the other cell types showed similar sensitivity (see Additional file 3: Figure S3, Additional file 4: Figure S4, Additional file 5: Figure S5). This result agrees with other published studies, where endothelial cells were found to be more sensitive than epithelial cells when exposed to cytotoxic compounds [36–38].

Of the four surface types, spNIPAM extracts had the highest average viability at this time point and concentration. This could possibly be explained by the uneven coverage of spNIPAM surfaces. SpNIPAM surfaces had the most uneven coating, with a lot of underlying surface exposed (as evidenced by the XPS measurements showed in Figure 3a). Therefore, they likely had the smallest amount of deposited pNIPAM, which could result in smaller amounts of pNIPAM (and other compounds that were involved in the deposition process) transferred to the extracts; therefore, fewer potential toxic effects.

### Concentration gradients

The higher sensitivity of BAECs was confirmed in concentration gradient experiments. Here, frpNIPAM and cpNIPAM were dissolved in regular cell culture media in concentrations ranging from 0.1 mg/mL to 10 mg/mL. See Additional file 6: Figure S6, Additional file 7: Figure S7, Additional file 8: Figure S8 for the results for the other three cell types. All these cell types showed average viability of around 100%, with small standard deviations for both cpNIPAM and frpNIPAM. BAECs proved to be more sensitive in this test as well.

**Figure 6:**
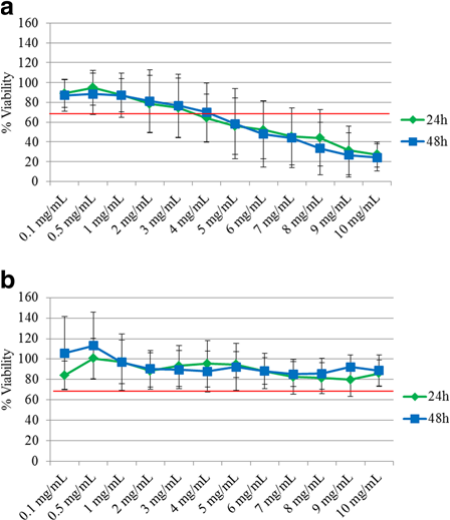
**MTS assay results for concentration gradient experiments with BAECs (a) on cpNIPAM/IPA surfaces, and (b) on frpNIPAM/IPA surfaces.** Red line indicates viability of 70%, below which a compound is considered to be cytotoxic. Corresponding figures for Veros, SMCs, and 3T3s can be found in supplemental information.

Figure 6 shows the result of concentration gradient experiments for BAECs exposed to cpNIPAM (a) and frpNIPAM (b). Cells exposed to frpNIPAM maintained average viability of 80% for both time points. However, these experiments yielded large standard deviations, with several values for single experiments dropping to or below 70%. CpNIPAM had even larger effect on BAECs. Starting at about 3/4 mg/mL, the viabilities for both time points (24 and 48 hours) decreased to reach values as low as 20% viability at the concentration of 10 mg/mL. Due to this unexpected result, this experiment was repeated 6 times (instead of the usual 3), to confirm that there indeed is a trend, and that the result is not due to infected cells or media. All six experiments showed a similar trend, with the viability starting to decrease between 3 and 5 mg/mL. The large standard deviation of the composite graph results from the differences between the single experiments, as the viabilities did vary slightly between the runs.

This variability is not explained by the presence of bacterial or other contaminants in the cpNIPAM test solution, as no decrease in viability, normal growth, and proliferation were observed in the other three cell types that were exposed to the same test solution. NMR of cpNIPAM was performed to confirm the identity and the extent of polymerization of this compound, which could affect the cytotoxicity. While confirming the identity of the polymer, the NMR spectrum showed presence of small amount of the monomer. The peaks corresponding to double bonds in the monomer were not visible on the NMR spectrum of frpNIPAM (see Additional file 9: Figure S9). The presence of small amounts of monomer could explain the results of the concentration gradient experiment. It would also account for the variability between the six experiments performed with cpNIPAM test solutions, as different amounts of the monomer could end up in the wells, resulting in different cellular toxicity. Since endothelial cells appeared to be most sensitive to the monomer, purification of the polymer before using it with this cell type would be recommended.

## Conclusions

In this work, we performed a comprehensive study of cytotoxicity of pNIPAM and pNIPAM-coated surfaces. We used commercially available pNIPAM as well as PNIPAM synthesized in our laboratory for the tests. These two polymers where used for the investigation of the cytotoxicity of pNIPAM using a concentration gradient test. We also generated four different pNIPAM-coated surfaces for the determination of the cytotoxicity of pNIPAM-coated surfaces: ppNIPAM, spNIPAM, frpNIPAM, and cpNIPAM. These surfaces were extensively tested with extracts and direct contact experiments. The cytotoxicity tests were performed with endothelial, epithelial, fibroblast, and smooth muscle cells.

We found that the NIPAM monomer in pure powdered form at 0.5 mg/mL is toxic to all tested cell types, except to fibroblasts at short term exposure. Endothelial and epithelial cells were the most sensitive to the monomer, while fibroblasts were the most resistant. Although initially the attachment and proliferation of fibroblast and smooth muscle cells was hindered on spNIPAM surfaces, long term experiments proved that all pNIPAM-coated surfaces were not cytotoxic to the four cell types evaluated in the direct contact test. Plating efficiency values did not show cytotoxic effects, except for the monomer, which was an expected result. Extract and concentration gradient experiments showed no cytotoxic effects when tested with epithelial, smooth muscle, and fibroblast cells. Endothelial cells showed increased sensitivity to extracts at the 100% concentration after 48 hour exposure. Concentration gradient experiments showed that endothelial cells were more sensitive to commercially available pNIPAM, which was a likely result of presence of some monomer. These results agree with other published findings, where endothelial cells were found to be more sensitive than epithelial cells.

Since it was discovered that cellular sensitivity to pNIPAM varies depending on cell type, it would be recommended to perform cytotoxicity testing on cell types previously unexposed to pNIPAM before using them with this polymer for research. Also, the purity of the polymer is essential, as demonstrated by the concentration gradient experiments. We also found that while cell viability on pNIPAM surfaces decreases when compared to controls, the viability also seems to be deposition type dependent, with sol–gel-based pNIPAM surfaces being the least biocompatible.

## Electronic supplementary material

Additional file 5: Figure S5: MTS assay results for culture of 3T3s in the presence of pNIPAM extracts. Red line indicates viability of 70%, below which a compound is considered to be cytotoxic. (PNG 92 KB)

Additional file 1: Figure S1: High resolution C1s spectra for (a) ppNIPAM, (b) spNIPAM, (c) frpNIPAM, and (d) cpNIPAM surfaces. (PNG 65 KB)

Additional file 2: Figure S2: LIVE/DEAD assay result for SMC, 3T3, BAEC, and Vero cells cultured on uncoated glass slides (controls). (PNG 773 KB)

Additional file 3: Figure S3: MTS assay results for culture of SMCs in the presence of pNIPAM extracts. Red line indicates viability of 70%, below which a compound is considered to be cytotoxic. (PNG 98 KB)

Additional file 4: Figure S4: MTS assay results for culture of Veros in the presence of pNIPAM extracts. Red line indicates viability of 70%, below which a compound is considered to be cytotoxic. (PNG 93 KB)

Additional file 6: Figure S6: MTS assay results for concentration gradient experiments with SMCs (a) on cpNIPAM/IPA surfaces, and (b) on frpNIPAM/IPA surfaces. Red line indicates viability of 70%, below which a compound is considered to be cytotoxic. (PNG 49 KB)

Additional file 7: Figure S7: MTS assay results for concentration gradient experiments with Veros (a) on cpNIPAM/IPA surfaces, and (b) on frpNIPAM/IPA surfaces. Red line indicates viability of 70%, below which a compound is considered to be cytotoxic. (PNG 48 KB)

Additional file 8: Figure S8: MTS assay results for concentration gradient experiments with 3T3s (a) on cpNIPAM/IPA surfaces, and (b) on frpNIPAM/IPA surfaces. Red line indicates viability of 70%, below which a compound is considered to be cytotoxic. (PNG 51 KB)

Additional file 9: Figure S9: NMR spectra of frpNIPAM (top, blue) and cpNIPAM (bottom, red). Red box indicates the peaks corresponding to hydrogens attached to double bonded carbons (indicative of the presence of monomer). (PNG 24 KB)
