# Supplementary figures and images for: Assessment of cytotoxicity of (N-isopropyl acrylamide) and Poly(N-isopropyl acrylamide)-coated surfaces
Source: Biointerphases. 2013 Aug 7;8(1):19. doi: 10.1186/1559-4106-8-19 (PMC3979476; doi:10.1186/1559-4106-8-19)

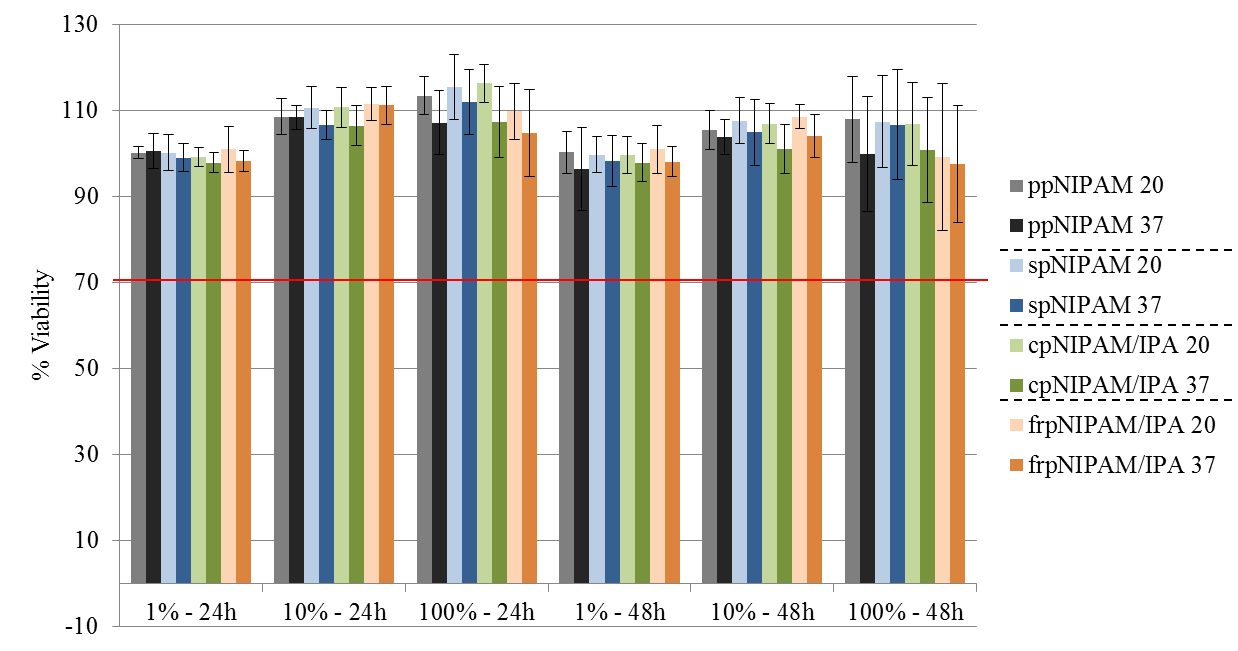

Supplement: Supplementary file 1 — Additional file 5: Figure S5: MTS assay results for culture of 3T3s in the presence of pNIPAM extracts. Red line indicates viability of 70%, below which a compound is considered to be cytotoxic. (PNG 92 KB) [file BJIOBN-000008-000019_1-s005.png]

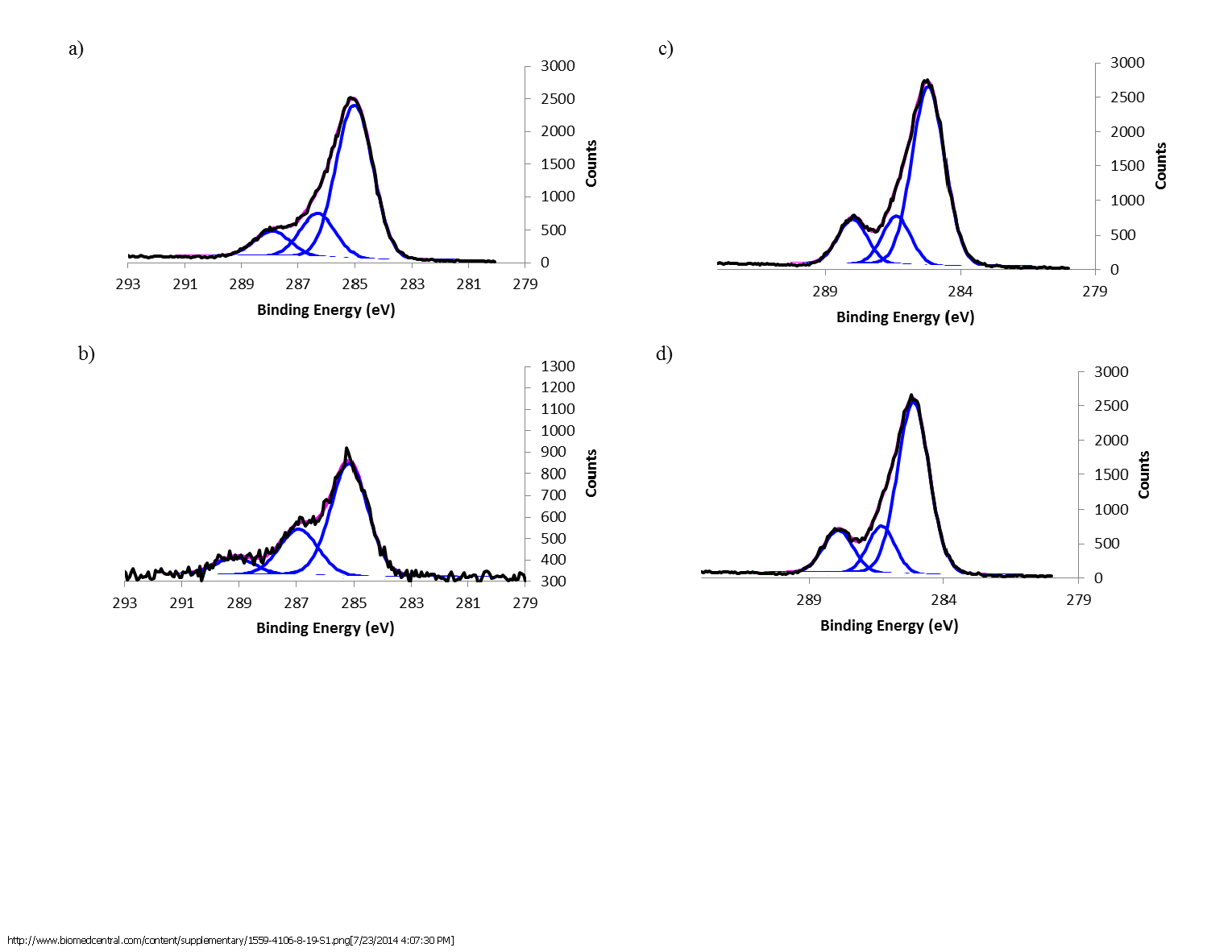

Supplement: Supplementary file 2 — Additional file 1: Figure S1: High resolution C1s spectra for (a) ppNIPAM, (b) spNIPAM, (c) frpNIPAM, and (d) cpNIPAM surfaces. (PNG 65 KB) [file BJIOBN-000008-000019_1-s001.png]

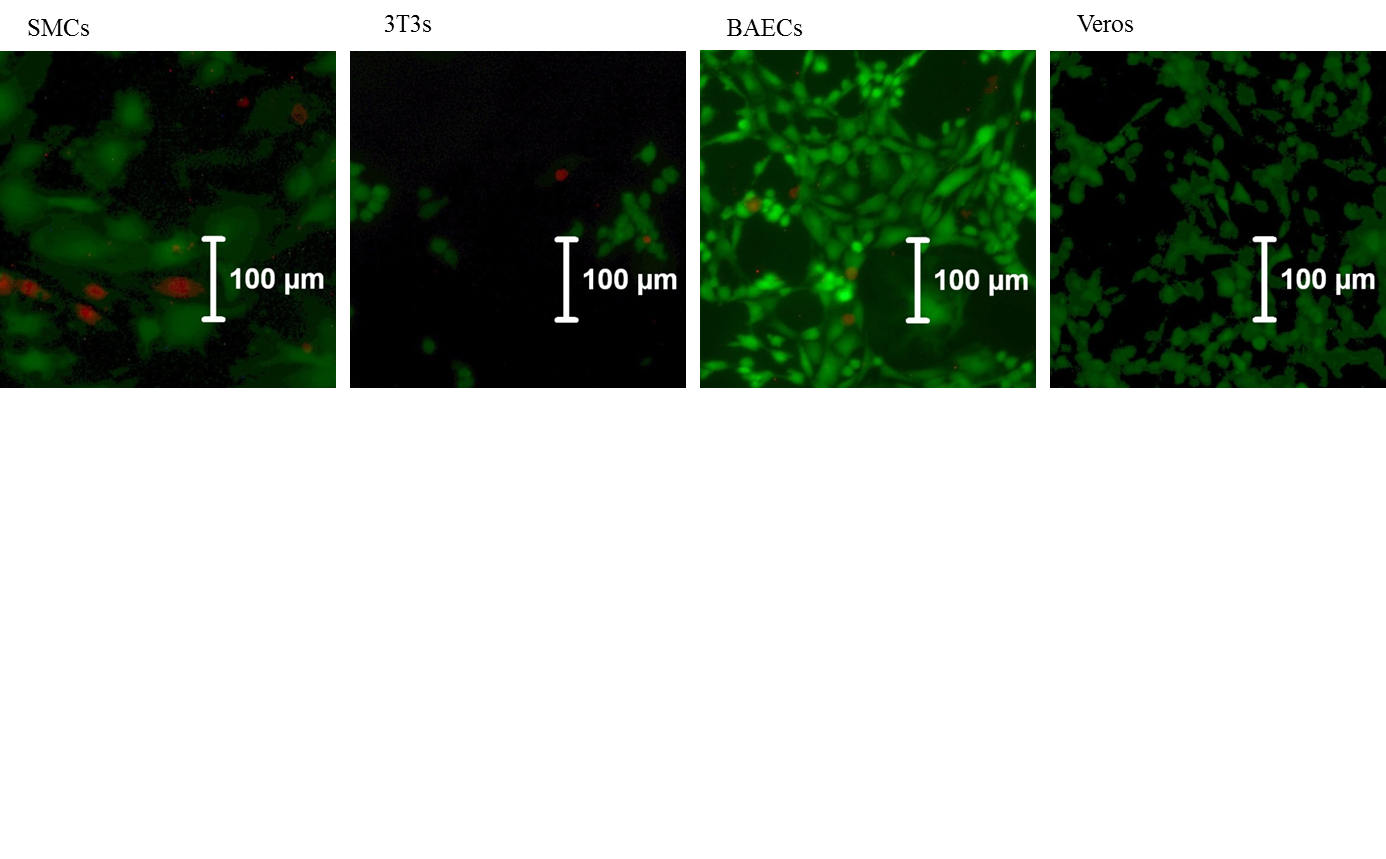

Supplement: Supplementary file 3 — Additional file 2: Figure S2: LIVE/DEAD assay result for SMC, 3T3, BAEC, and Vero cells cultured on uncoated glass slides (controls). (PNG 773 KB) [file BJIOBN-000008-000019_1-s002.png]

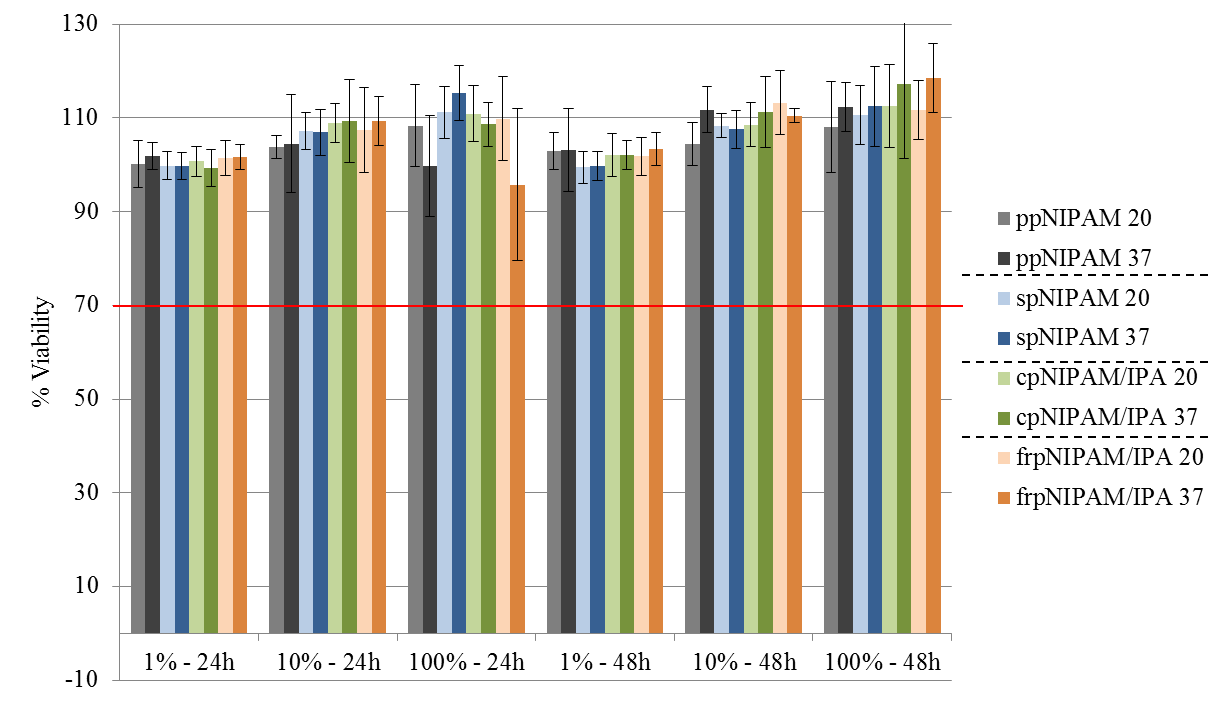

Supplement: Supplementary file 4 — Additional file 3: Figure S3: MTS assay results for culture of SMCs in the presence of pNIPAM extracts. Red line indicates viability of 70%, below which a compound is considered to be cytotoxic. (PNG 98 KB) [file BJIOBN-000008-000019_1-s003.png]

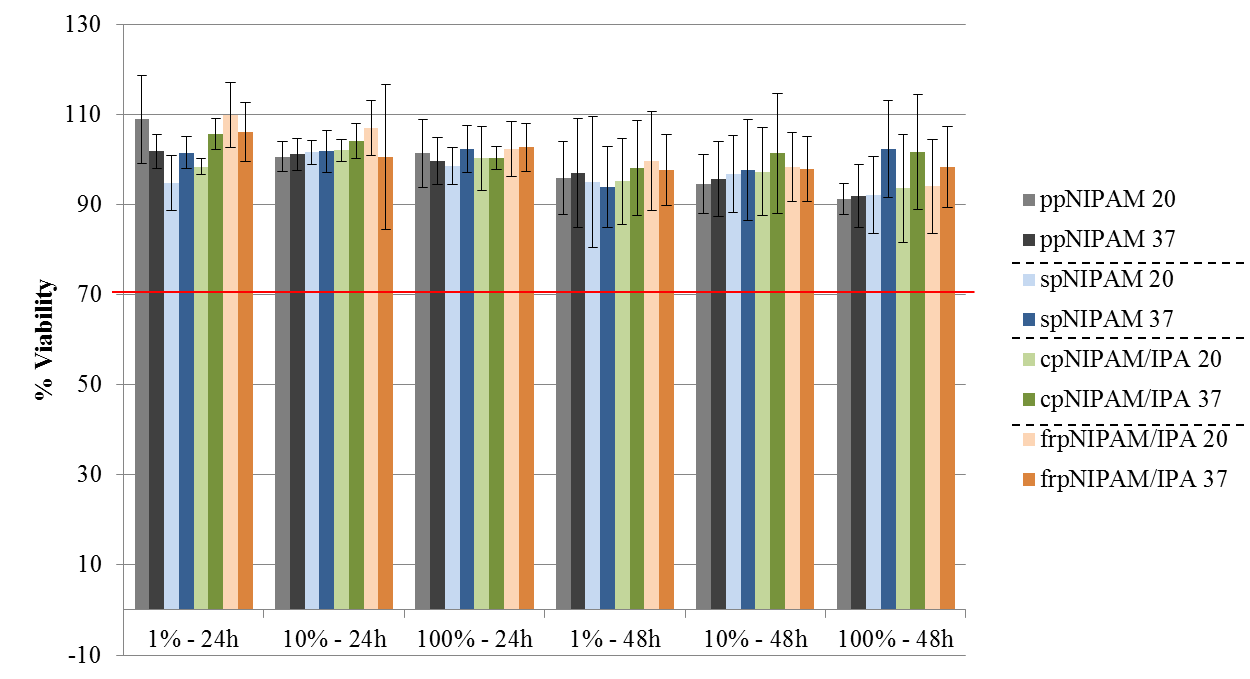

Supplement: Supplementary file 5 — Additional file 4: Figure S4: MTS assay results for culture of Veros in the presence of pNIPAM extracts. Red line indicates viability of 70%, below which a compound is considered to be cytotoxic. (PNG 93 KB) [file BJIOBN-000008-000019_1-s004.png]

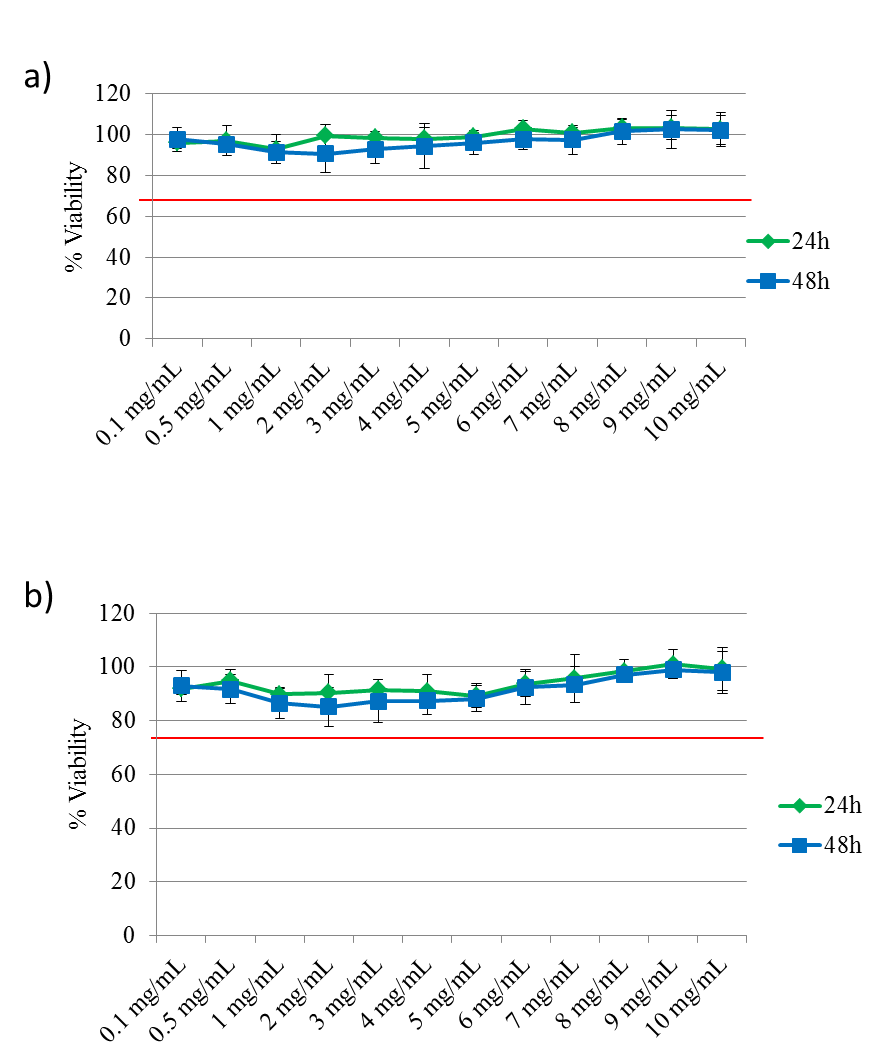

Supplement: Supplementary file 6 — Additional file 6: Figure S6: MTS assay results for concentration gradient experiments with SMCs (a) on cpNIPAM/IPA surfaces, and (b) on frpNIPAM/IPA surfaces. Red line indicates viability of 70%, below which a compound is considered to be cytotoxic. (PNG 49 KB) [file BJIOBN-000008-000019_1-s006.png]

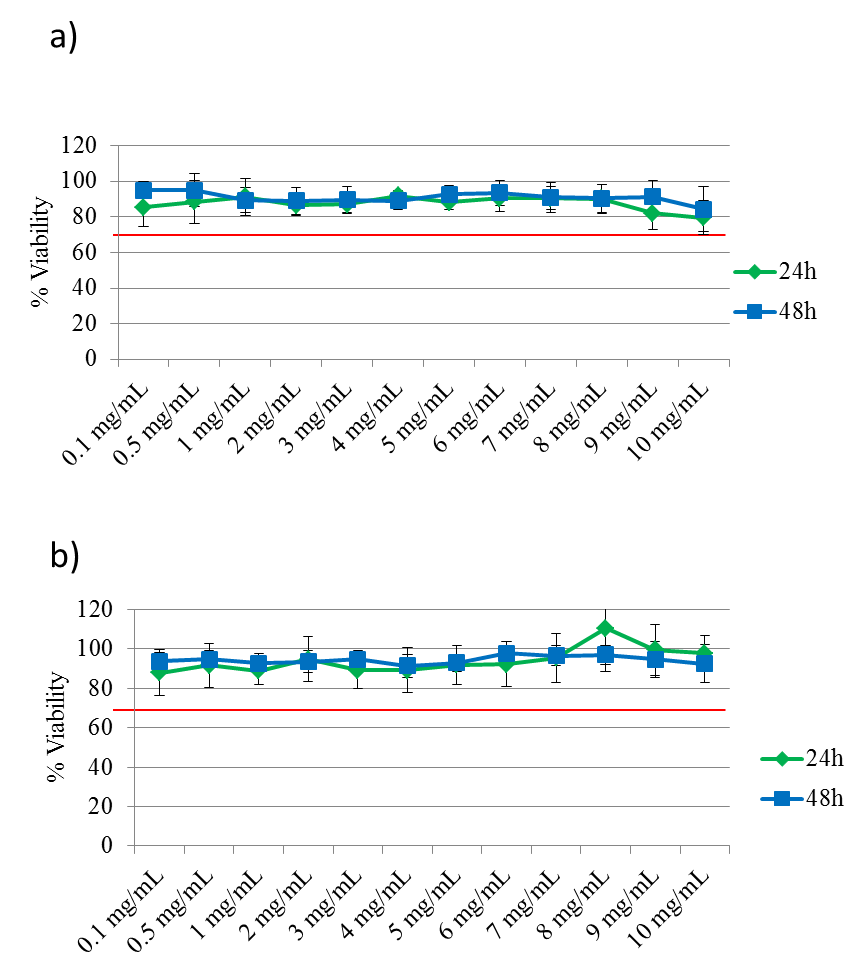

Supplement: Supplementary file 7 — Additional file 7: Figure S7: MTS assay results for concentration gradient experiments with Veros (a) on cpNIPAM/IPA surfaces, and (b) on frpNIPAM/IPA surfaces. Red line indicates viability of 70%, below which a compound is considered to be cytotoxic. (PNG 48 KB) [file BJIOBN-000008-000019_1-s007.png]

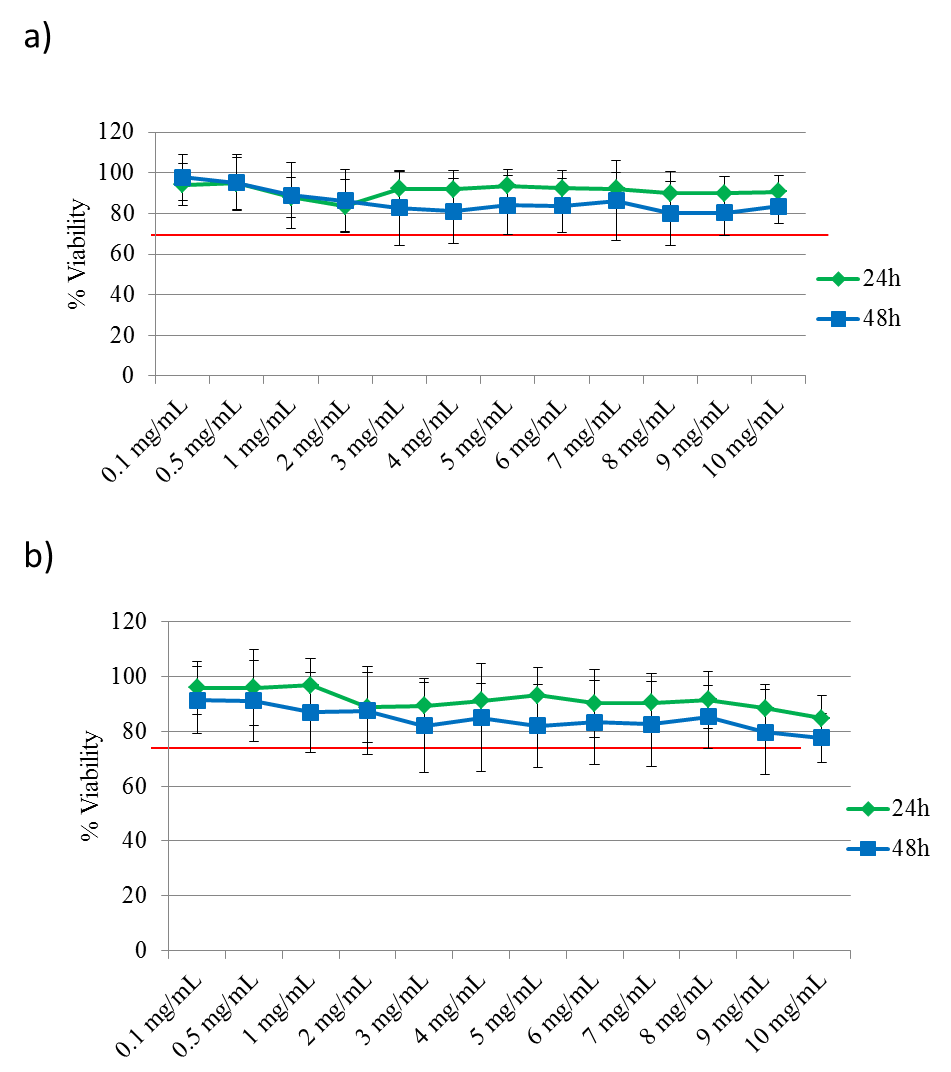

Supplement: Supplementary file 8 — Additional file 8: Figure S8: MTS assay results for concentration gradient experiments with 3T3s (a) on cpNIPAM/IPA surfaces, and (b) on frpNIPAM/IPA surfaces. Red line indicates viability of 70%, below which a compound is considered to be cytotoxic. (PNG 51 KB) [file BJIOBN-000008-000019_1-s008.png]

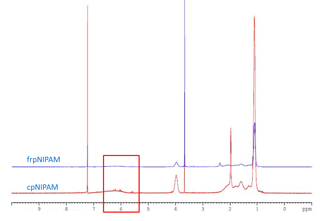

Supplement: Supplementary file 9 — Additional file 9: Figure S9: NMR spectra of frpNIPAM (top, blue) and cpNIPAM (bottom, red). Red box indicates the peaks corresponding to hydrogens attached to double bonded carbons (indicative of the presence of monomer). (PNG 24 KB) [file BJIOBN-000008-000019_1-s009.png]
